# Plant Genetic Background Increasing the Efficiency and Durability of Major Resistance Genes to Root-knot Nematodes Can Be Resolved into a Few Resistance QTLs

**DOI:** 10.3389/fpls.2016.00632

**Published:** 2016-05-10

**Authors:** Arnaud Barbary, Caroline Djian-Caporalino, Nathalie Marteu, Ariane Fazari, Bernard Caromel, Philippe Castagnone-Sereno, Alain Palloix

**Affiliations:** ^1^INRA, University of Nice Sophia Antipolis, CNRS, UMR 1355-7254, Institut Sophia AgrobiotechSophia Antipolis, France; ^2^INRA, UR1052, Génétique et Amélioration des Fruits et LégumesMontfavet, France

**Keywords:** *Capsicum annuum*, *Meloidogyne* spp., quantitative resistance, major resistance, resistancedurability

## Abstract

With the banning of most chemical nematicides, the control of root-knot nematodes (RKNs) in vegetable crops is now based essentially on the deployment of single, major resistance genes (*R*-genes). However, these genes are rare and their efficacy is threatened by the capacity of RKNs to adapt. In pepper, several dominant *R-*genes are effective against RKNs, and their efficacy and durability have been shown to be greater in a partially resistant genetic background. However, the genetic determinants of this partial resistance were unknown. Here, a quantitative trait loci (QTL) analysis was performed on the F_2:3_ population from the cross between Yolo Wonder, an accession considered partially resistant or resistant, depending on the RKN species, and Doux Long des Landes, a susceptible cultivar. A genetic linkage map was constructed from 130 F_2_ individuals, and the 130 F_3_ families were tested for resistance to the three main RKN species, *Meloidogyne incognita, M. arenaria*, and *M. javanica*. For the first time in the pepper-RKN pathosystem, four major QTLs were identified and mapped to two clusters. The cluster on chromosome P1 includes three tightly linked QTLs with specific effects against individual RKN species. The fourth QTL, providing specific resistance to *M. javanica*, mapped to pepper chromosome P9, which is known to carry multiple NBS–LRR repeats, together with major *R-*genes for resistance to nematodes and other pathogens. The newly discovered cluster on chromosome P1 has a broad spectrum of action with major additive effects on resistance. These data highlight the role of host QTLs involved in plant-RKN interactions and provide innovative potential for the breeding of new pepper cultivars or rootstocks combining quantitative resistance and major *R-*genes, to increase both the efficacy and durability of RKN control by resistance genes.

## Introduction

Root-knot nematodes (RKNs), *Meloidogyne* spp., are major plant pathogens worldwide. These extremely polyphagous endoparasites can infest more than 5,500 plant species, including many field and greenhouse crops (Goodey et al., 1965). Since the banning of most chemical nematicides, due to environmental and public health issues, resistant cultivars have become an increasing important weapon against these pests. This method efficiently controls RKN populations, and is economically sustainable, innocuous to health and environment-friendly. RKN resistance is generally mediated by single, major resistance genes (*R*-genes), which can easily be introgressed into cultivars through back-crossing and phenotypic or marker-assisted selection (MAS). For this reason, major *R*-genes are widely used in the breeding of RKN-resistant cultivars and/or rootstocks. However, their efficacy is threatened by the capacity of RKNs to adapt. Indeed, *R*-genes apply a selective pressure on nematode populations, increasing the risk of virulent nematode populations emerging (Castagnone-Sereno, 2006), and this greatly limits their use. Several management strategies have been developed, to prevent the breakdown of resistance by pathogens. Most of these approaches are based on spatiotemporal management of the deployment of *R*-genes: (i) alternation of different *R*-genes in the crop rotation, (ii) use of mixtures of cultivars with different *R*-genes, or (iii) pyramiding, the introduction of several *R*-genes into the same cultivar (Kiyosawa, 1982; Mundt, 2002; Pink, 2002). The use of such strategies requires several *R*-genes to be available, with no emergence of cross-virulent pathogens. Recent experimental studies have shown that the pyramiding of *R*-genes is the best method for promoting effective, durable RKN resistance in pepper (Djian-Caporalino et al., 2014).

Several recent studies, on very different pathosystems, have shown that the genetic background of the plant greatly influences *R*-gene efficiency, potentially slowing the adaptation of pathogen populations to *R*-gene-carrying cultivars (Palloix et al., 2009; Brun et al., 2010; Fournet et al., 2013; Barbary et al., 2014). In some pathosystems, this greater durability has been shown to result from quantitative trait loci (QTLs), which slow the selection of variants virulent against the *R*-gene and decrease the size of the pathogen population (e.g., Quenouille et al., 2014). Plant genetic background is rarely considered in breeding programs for RKN resistance, despite its contribution to *R*-gene efficiency and durability. Indeed, breeding for resistance with QTLs is more complex and costly than the use of *R*-genes. In particular, the introgression of QTLs into elite cultivars must not impair other agronomically important crop traits, such as yield, quality criteria and adaptation, or other physiological characteristics.

In pepper (*Capsicum annuum* L.), several dominant *R*-genes, the *Me* genes and the *N* gene, have been characterized in detail (Hare, 1956; Hendy et al., 1985; Djian-Caporalino et al., 1999; Thies and Fery, 2000). These genes map to a genetic cluster on pepper chromosome P9 (Djian-Caporalino et al., 2007; Fazari et al., 2012). Three of these genes, *Me3, Me1*, and *N*, are routinely used in breeding programs. These genes are effective against a wide range of RKN species, including *M. arenaria, M. incognita*, and *M. javanica*, the most common species in temperate and tropical areas. They differ in their mode of action: *Me3* and *Me1* are stable at high temperature (Djian-Caporalino et al., 1999), whereas the efficacy of *N* is temperature-dependent (Thies and Fery, 1998). In addition, *Me3* and *Me1* differ in the spatiotemporal location of the resistance response triggered by a nematode attack. *Me3* triggers an early hypersensitive response in the root epidermis at the nematode penetration site, whereas *Me1* triggers a later response in the root vascular cylinder (Bleve-Zacheo et al., 1998; Pegard et al., 2005). It was long thought that there was a relationship between the mode of action of these *R*-genes and their durability, as the emergence of virulent populations has been reported only for *N* and *Me3* (Castagnone-Sereno et al., 1996; Thies, 2011). However, the risk of *Me1*-virulent RKN populations emerging and of the development of multi-virulent populations might increase with the extensive deployment of these resistance genes in agriculture. The efficacy of these genes has been shown to be higher when they are introgressed into a partially resistant background and lower if they are introgressed into a highly susceptible background (Barbary et al., 2014). These same genetic backgrounds were also previously shown to affect the durability of field resistance (Djian-Caporalino et al., 2014). However, no quantitative resistance loci that could be combined with major genes to increase the efficacy and durability of genetic RKN control have yet been identified in the pepper germplasm.

We report here a QTL analysis dissecting the genetic backgrounds previously shown to modulate the efficacy and durability of resistance. An F_2:3_ progeny derived from a cross between a partially resistant (Yolo Wonder, YW) and a highly susceptible (Doux Long des Landes, DLL) pepper inbred line was tested for quantitative resistance to the three main RKN species (*M. incognita, M. arenaria*, and *M. javanica*). Four new major QTLs were mapped to two separate clusters. The first, containing one QTL, colocalized with the cluster of *Me* genes on pepper chromosome P9. The second included three QTLs against the three *Meloidogyne* species located on pepper chromosome P1. This new cluster could potentially be used for innovative breeding strategies to increase *R*-gene efficacy and durability for the control of RKNs.

## Materials and Methods

### Plant Material

**FIGURE 1:**
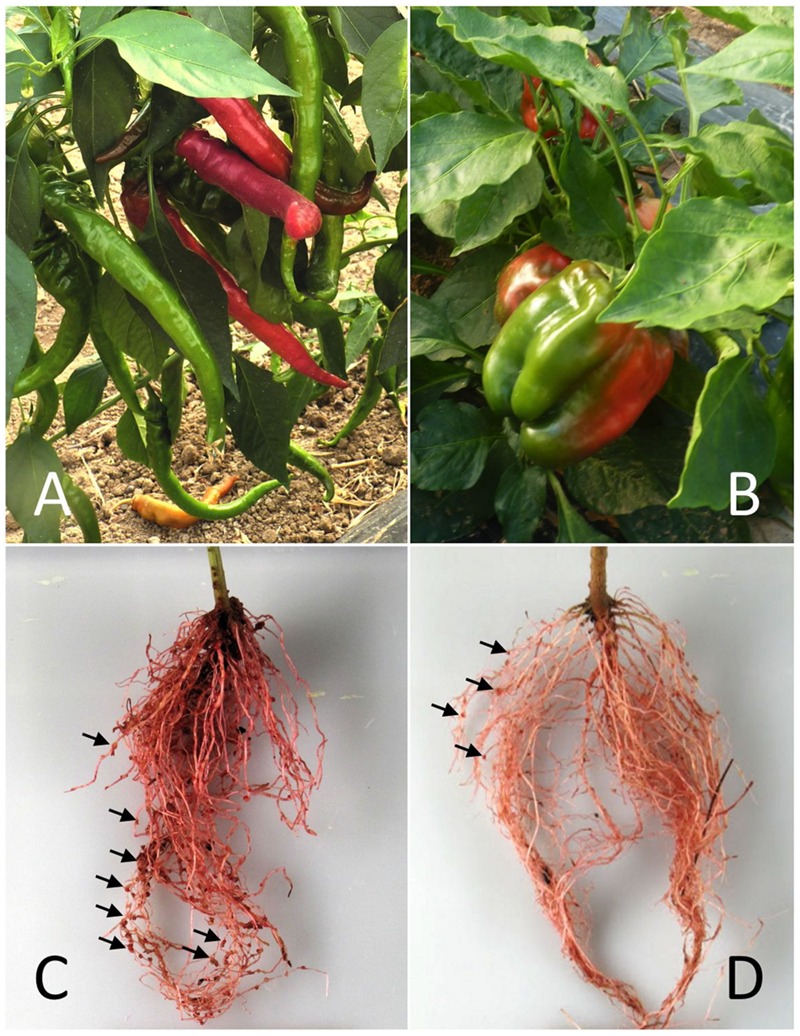
**The pepper/*Meloidogyne incognita* pathosystem used in this study.** The susceptible and resistant pepper cultivars Doux Long des Landes (DLL) **(A)** and Yolo Wonder (YW) **(B)** and their respective root systems **(C,D)** 6 weeks after inoculation with the nematodes. Egg masses (EMs; arrows) were stained with cold eosin (red).

A population of 130 F_2:3_ families derived from a cross between YW and DLL was used. These pepper cultivars were selected from the INRA pepper germplasm collection at Avignon, France (CRB-Leg, INRA-GAFL), on the basis of their different levels of resistance to nematode species. DLL is highly susceptible to the three main RKN species: *M. arenaria, M. javanica*, and *M. incognita*. YW is partially resistant (i.e., low-level symptoms) to *M. incognita* (**Figure 1**), totally resistant to *M. javanica* and has variable levels of resistance to *M. arenaria* (i.e., totally or partially resistant), depending on the RKN isolate considered (Djian-Caporalino et al., 1999). A single F_1_ hybrid plant was self-pollinated to generate 130 F_2_ plants, which were used to construct the genetic map. The 130 F_2_ plants were self-pollinated to generate 130 F_3_ families, which were used to assess disease resistance.

### Nematode Isolates

Three RKN species were used for resistance tests in controlled conditions. The first, *M. incognita* (Morelos isolate), causes disease on DLL, which is susceptible, whereas YW is partially resistant. The other two species used were *M. arenaria* (Marmande isolate) and *M. javanica* (Avignon isolate). DLL is susceptible and YW is highly resistant to these two species (Djian-Caporalino et al., 1999). These nematodes were obtained from the INRA *Meloidogyne* collection maintained at Institut Sophia Agrobiotech in Sophia Antipolis, France. These three *Meloidogyne* species have a mitotic parthenogenetic mode of reproduction. We therefore considered all the second-stage juveniles (J2s) hatching from a single egg mass to constitute a clonal line. Before multiplication, these isolates were specifically identified on the basis of isoesterase electrophoresis (Dalmasso and Berge, 1978) or sequence characterized amplified region (SCAR) PCR (Zijlstra et al., 2000).

### DNA Extraction, Genotyping of Molecular Markers, and Linkage Map

Genomic DNA was isolated from the young leaves of both parents, the F_1_ and the individuals of the mapping population, as described by Fulton et al. (1995). After RNAse treatment, the concentration and purity of DNA were assessed with a NanoDrop 2000 spectrophotometer (Thermo Scientific), and the final DNA concentration was adjusted to 20 ng/μL for PCR.

The F_2_ mapping population was genotyped with 58 markers previously used in other populations: one B94 SCAR marker (Djian-Caporalino et al., 2007), 13 simple sequence repeat (SSR) markers previously mapped in pepper (Alimi et al., 2013), 44 single-nucleotide polymorphism (SNP) markers from Nicolaï et al. (2012). In addition, 272 new SNP markers were provided by Syngenta Seeds. We used these markers to construct a genetic linkage map with Mapmaker software version 3.0b (Lander et al., 1987), using a LOD score threshold of 3.0 and a maximum recombination fraction of 0.3. Distances between markers were calculated with the Kosambi mapping function. For each linkage group (LG), marker order was checked with the ‘ripple’ command and markers were retained only if the LOD score value was greater than 2.0. The LGs were assigned to pepper chromosomes on the basis of the positions of SSR and SNP markers common to the genetic maps for pepper published by Alimi et al. (2013) and Quenouille et al. (2014).

### Experimental Procedures for Evaluating Nematode Resistance

Resistance was assessed on the F_3_ progenies. For each RKN isolate, 16 F_3_ seeds per F_2:3_ family were sown individually in 9 cm plastic pots containing steam-sterilized sandy soil covered with a 1 cm layer of loam. F_3_ plants were split evenly (i.e., eight plants per F_3_) between two growth chambers maintained at 24°C (±2°C) with a 12-h light12-h dark cycle and a relative humidity of 60–70%. Parental lines, the F_1_ progeny and two resistant controls (HD149 and HD330) were included in the experimental design. The 130 F_2:3_ families and controls were randomly arranged within each growth chamber. Six to seven-week-old plants (4–6 true leaves) were each inoculated with 500 freshly hatched J2s suspended in water, for experiments with *M. arenaria* and *M. javanica*, and with 1,000 J2s suspended in water for experiments with *M. incognita*. This difference in inoculum was based on the behavior of YW with respect to the species used (resistant or partially resistant). It has been shown that a higher inoculum density is required to reveal the differences between the partially resistant parent (YW) and the highly susceptible parent (DLL). J2s were obtained in a mist chamber, from previously inoculated susceptible tomato roots (cultivar Saint Pierre). Six to seven weeks after inoculation (i.e., a period long enough for completion of the nematode life cycle), plants were harvested, carefully washed individually with tap water and frozen at –20°C until scoring. Before analysis, the roots were thawed and stained by incubation for 10 min in a cold aqueous solution of eosin (0.1 g/l water), for the specific staining of egg masses (EMs). The roots were rinsed and examined under a magnifying glass. The number of EMs was counted for each plant and the median number of EMs per plant for each F_3_ family (and for the control genotype) was determined, for estimation of the phenotypic value of each F_2_.

### Statistical Analyses

R software^1^ was used for descriptive statistics. Analyses of variance (ANOVA) were carried out for each phenotypic trait (i.e., for resistance to each RKN species), to estimate genotypic/environmental effects. For each phenotypic trait, narrow-sense heritability (*h*^2^) was estimated with the formula h^2^ = 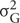/(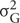
+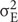/n), where 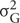 corresponds to the genotypic variance and 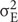 to the environment variance (including block, interaction, and error effects) and *n* is the number of replicates per F_2:3_ family (two growth rooms). Additive and dominance effects were calculated as described by Stuber et al. (1987). The normality of phenotype distributions was assessed with Shapiro–Wilks tests (α = 0.05).

Quantitative trait loci analyses were performed with the R/qtl package of R software (Broman et al., 2003). QTLs were detected by regression analysis, SIM, CIM, and non-parametric interval mapping (model = “np” in the R/qtl package) for the non-Gaussian phenotype distributions, although the residues were normal, making it possible to carry out a regression analysis to estimate additive and *R*^2^ values. All the methods yielded similar results (the same QTLs at the same positions; data not shown), although the QTL peaks were slightly less sharp with the non-parametric procedure. A permutation test was performed with 1,000 replicates to determine the genome-wide LOD threshold empirically at the 5% probability level, for each phenotypic trait individually. The LOD threshold was estimated at 3.6 for the three traits. For each QTL, the confidence interval (CI) was defined as a 2-LOD drop-off around the maximum LOD score. *R*^2^ coefficients were calculated with the ‘fitqtl’ function of R/qtl.

## Results

### Linkage Map

Four of the 330 markers tested on the F_2_ mapping population remained unlinked. The genetic linkage map was therefore constructed with 326 markers: 13 SSRs, 312 SNPs, and 1 SCAR. Markers displaying significant deviation from the expected Mendelian ratio of 1:2:1 were retained (indicated by asterisks in Supplementary Data 1). The map comprised 12 LGs, corresponding to the 12 pepper chromosomes (P1–P12) and covering an overall length of 1436 cM (Supplementary Data 1).

### Segregation of Resistance to the Three RKN Species

**FIGURE 2:**
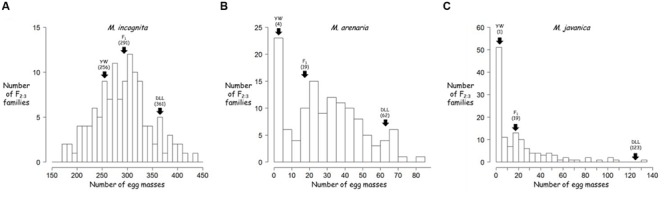
**Frequency distribution of the resistance to different root-knot nematode (RKN) species of the pepper F_2:3_ families: **(A)***M. incognita*; **(B)***M. arenaria*; and **(C)***M. javanica*.** Arrows indicate the position of each parent and the F_1_ in the phenotypic distribution, with their values indicated in brackets. YW, Yolo Wonder; DLL, Doux Long des Landes; F1, (YW × DLL).

The frequency distributions of resistance to *M. incognita, M. arenaria*, and *M. javanica* in the F_2:3_ families are shown in **Figure 2**. For *M. incognita*, the effects of both genotype and block were significant (*p*-value = 0.000653 and *p*-value < 2.00*^e^*^-16^, respectively) as revealed by ANOVA. The regression of F_3_ values from block 1 over block 2 was significant (R_Pearson_ = 0.32, *p* = 0.00027) with higher values in block 1. Individual EM data were therefore adjusted by multiplying the data from block 2 by the regression coefficient (value: 1.4). This linear adjustment removed the block effect and the data were pooled for further analyses. The phenotypes of the control pepper lines were consistent with the expected results (**Table 1**): YW presented a lower infestation rate than DLL (mean of 250 and 330 EMs/plant, respectively). The F_1_ progeny was skewed toward DLL (319 EMs/plant), with a *d*/*a* ratio of –0.72, indicating additive to partly dominant inheritance in favor of susceptibility. A Shapiro–Wilk test showed that the values for the F_2:3_ families were normally distributed (*W* = 0.99, *p*-value = 0.2029; **Figure 2A**). The *h*^2^ value was 0.48.

**Table 1:** Summary of root-knot nematode (RKN) reproduction capacity in the parental lines, F1 and F_2:3_ progeny (130 F_2:3_ families) from the pepper cross (YW × DLL), for different RKN species.

RKN species	Parents		F_1_	F_2:3_ families				
	YW	DLL		Maximum	Minimum	Mean	*SD*	*h*^2^
*Meloidogyne incognita*	250	330	319	519	172	332	67	0.48
*Meloidogyne arenaria*	5	69	14	82	0	25	21	0.76
*Meloidogyne javanica*	1	131	17	132	0	17	26	0.87

*Reproduction capacities were evaluated as the median number of egg masses (EMs) on 16 plants for each genotype. YW, Yolo Wonder (resistant or partially resistant genotype); DLL, Doux Long des Landes (susceptible genotype); F_*1*_, hybrid F_*1*_; SD, standard deviation; *h*^*2*^, broad-sense heritability (h^2^ = 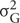/(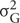
+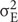/n)).*

For the experiment with *M. arenaria*, ANOVA revealed a significant effect of genotype (*p*-value = 3.26*^e^*^-15^), but no significant block effect (*p*-value = 0.0805). YW and DLL were resistant and susceptible (mean values of 5 and 69 EMs/plant, respectively), as expected (**Table 1**). The F_1_ phenotype was skewed toward YW (14 EMs/plant), with a *d*/*a* ratio of 0.72, indicating that resistance was additive to partly dominant in favor of resistance. The values for the F_2:3_ families were not normally distributed, as confirmed by a Shapiro–Wilk test (*W* = 0.92, *p*-value = 8.63*^e^*^-07^; **Figure 2B**). The distribution was skewed toward resistance. Neither logarithmic nor square-root transformation resulted in normality (data not shown). Resistance to *M. arenaria* was highly heritable, as *h*^2^ was 0.76.

For the experiment with *M. javanica*, ANOVA showed a significant effect of genotype (*p*-value < 2.00*^e^*^-16^) but no significant block effect (*p* > 0.183). YW was highly resistant and DLL was susceptible, as expected (means of 1 and 131 EMs/plant, respectively; **Table 1**). The F_1_ displayed an intermediate phenotype, with skewing toward YW (mean of 17 EMs/plant), with a *d*/*a* ratio equal to 0.75, indicating that resistance was mostly dominant, but slightly additive. The Shapiro–Wilk test indicated that the data for the F_2:3_ families were not normally distributed (*W* = 0.66, *p*-value = 2.80*^e^*^-15^; **Figure 2C**). Neither logarithmic nor square-root transformation yielded normality. Heritability was high for resistance to *M. javanica* (*h*^2^ = 0.87).

### Mapping QTLs for Resistance to the Three *Meloidogyne* Isolates

**FIGURE 3:**
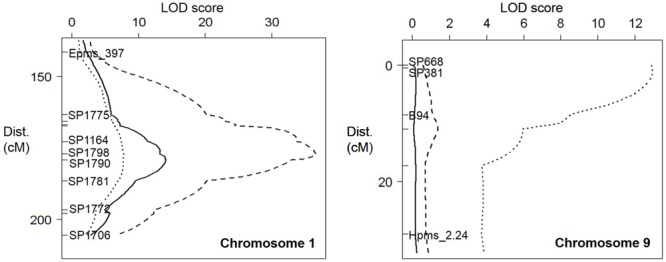
**Quantitative trait loci (QTLs) against *M. incognita* (solid line), *M. arenaria* (dashed line), and *M. javanica* (dotted line) on pepper chromosome 1 **(Left)** and chromosome 9 **(Right)**.** On the left of each linkage group (LG), distances in centimorgans, flanking markers, and the marker closest to the resistance factors are indicated.

Simple interval mapping (SIM), composite interval mapping (CIM) and non-parametric (“np” or Kruskal–Wallis) analysis were performed to identify QTLs for resistance. However, as CIM and non-parametric analyses did not improve QTL detection, only the results for SIM are shown. Only one QTL for resistance to *M. incognita* was detected on pepper chromosome P1, with a LOD_max_ at 179.2 cM and an *R*^2^ value of 40.9, corresponding to 85% of the heritability (*h^2^* = 0.48; **Figure 3**; **Table 2**). This QTL was named *Minc-P1*. The *d*/*a* ratio of –0.38 at the closest marker (SP1790) indicates a mostly additive effect of this QTL, with a partial dominance effect for susceptibility.

**Table 2:** Quantitative trait loci (QTLs) for resistance to the different RKN species in the pepper F_2:3_ progeny.

RKN species	QTL	Chr.^a^	Location (cM)	Closest marker	LOD	CI^b^	Additive effect (a)	Dominance effect (d)	*d*/*a* ratio	*R*^2^
*M. incognita*	*Minc-P1*	1	179.2	SP1790	14.1	173.4–184.0	–55.7	21.1	–0.38	40.9
*M. arenaria*	*Mare-P1*	1	177.0	SP1798	36.6	173.4–179.0	–24.5	–6.8	0.28	73.8
*M. javanica*	*Mjav-P1*	1	178.0	SP1798	7.7	163.9–190.5	–14.2	–4.1	0.29	31.9
	*Mjav-P9*	9	1.0	SP381	12.9	0.0–6.4	–19.1	–13.9	0.73	52.4

*^a^Chromosome. ^b^CI: Confidence interval, defined as a LOD-2 drop-off around the maximum LOD score. Negative values for additive (a) or dominance (d) effects indicate that the allele from the resistant parent decreases the phenotype value.*

Similarly, only one QTL for resistance to *M. arenaria* was detected and mapped to chromosome P1 (*Mare-P1*) with a LOD_max_ at 177.0 cM, close to *Minc-P1*. This QTL, with an *R*^2^ value of 73.8 (97% of the *h*^2^ value) and a *d*/*a* ratio of 0.28, acts as a major additive QTL, with a weak dominance effect in favor of resistance.

Two QTLs for resistance to *M. javanica* were detected. The first, *Mjav-P1*, was located on chromosome P1 with a LOD_max_ at 178.0 cM, an *R*^2^ of 31.9 and a *d*/*a* ratio of 0.29, indicating a mostly additive effect. The second QTL, *Mjav-P9*, was detected on the distal part of chromosome P9 (closest marker SP381) with an *R*^2^ of 52.4 and a *d*/*a* of 0.73, indicating a mostly dominant effect in favor of resistance. Together, *Mjav-P1* and *Mjav-P9* explained 61.2% of the phenotypic variance, corresponding to 71% of the *h*^2^ value.

All the resistance-conferring alleles at these four QTLs originated from the partially resistant parent YW.

### Looking for Recombinant Individuals Within the P1 QTL Cluster

F_2_ individuals with recombinant genotypes for the QTL cluster on chromosome P1 were surveyed, focusing on their genotypes for the markers and the phenotypes of their F_3_ progenies (homogeneous resistant, homogeneous susceptible, or segregating). Four F_2_ individuals were found to be recombinant for the alleles at the markers within the P1 QTL cluster (recombination between SP1790 and SP1798). Two F_2:3_ progenies for these individuals clearly corresponded to phenotypic recombinants in terms of their resistance to *M. arenaria* and *M. incognita*, as attested by the phenotypes of the F_3_ progenies (**Table 3**). Resistance to *M. javanica* was less informative, probably due to the major effect of the second locus *Mjav-P9*.

**Table 3:** Recombinant genotypes within the cluster of QTLs on chromosome P1 containing *Minc-P1* and *Mare-P1*.

	Alleles (YW versus DLL) at the markers				Phenotype^∗^ (EMs)	
F_2:3_ family	SP1164	SP1798	SP1790	SP1781	*M. arenaria*	*M. incognita*
17	YW/YW	YW/YW	YW/DLL	YW/DLL	R (3)	He (310)
22	YW/DLL	YW/DLL	DLL/DLL	m.d.	He (13)	S (478)
29	DLL/DLL	DLL/DLL	YW/DLL	YW/DLL	S (48)	S (398)
66	m.d.	YW/DLL	DLL/DLL	DLL/DLL	S (37)	He (306)

*Alleles at the marker: YW, Yolo Wonder (resistant allele); DLL, Doux Long des Landes (susceptible allele); m.d., missing data. ^∗^Observed segregation in the F_*3*_ family: R, all F_*3*_ plants resistant, S, all F_*3*_ plants susceptible, He, segregation of susceptible, and resistant plants within the F_*3*_ family. The value in brackets is the median number of EMs per plant for each F_*2:3*_ family.*

## Discussion

The pepper genetic map constructed in this study with 130 F_2_ plants from the cross between YW and DLL comprised 12 LGs, consistent with the known number of chromosomes in pepper, and it covered a total length of 1436 cM, consistent with previous maps for pepper (Lefebvre et al., 2002; Paran et al., 2004; Wu et al., 2009). However, as only a few of the previously used markers proved to be polymorphic between the parental lines YW and DLL, new SNPs had to be developed to complete the map. The sequences supporting the SNPs targeting the QTLs are provided in Supplementary Data 2. This new mapping population was developed because it was shown in a previous study that *R*-genes are more effective and durable against RKN attacks when introgressed into the YW genetic background than when introgressed into the DLL genetic background (Barbary et al., 2014; Djian-Caporalino et al., 2014). This difference is thought to result from the partial resistance alleles carried by YW, which seem to protect the *R*-genes. This new map was constructed to identify these resistance factors and associated molecular markers, which should constitute valuable resources for further MAS.

The 130 F_2:3_ [YW × DLL] families were tested against the three main RKN species, *M. incognita, M. arenaria, and M. javanica.* The QTL analyses identified four new major QTLs affecting reproductive capacity, located in two separate clusters. No minor-effect QTL was detected, and the phenotypic variance explained by the major QTLs for each resistance trait closely fitted the *h*^2^ values (71–97%), indicating that almost all the genetic variance was explained by these major QTLs. Three of these QTLs were grouped on pepper chromosome P1, with overlapping CIs, in a 30 cM region. The YW alleles at these QTLs each confer resistance to a single species of RKN: *M. incognita, M. arenaria*, or *M. javanica*. These are the first QTLs conferring resistance to RKNs to have been detected in pepper, but QTLs conferring resistance to *Meloidogyne* spp., have already been mapped in soybean (Li et al., 2001), cotton (Shen et al., 2006), and cowpea (Muchero et al., 2009). This is also the first report of nematode resistance factors mapping to a genomic location other than chromosome P9 in pepper (i.e., on chromosome P1). All the RKN *R*-genes previously identified in pepper mapped to a cluster on P9 (Djian-Caporalino et al., 2007; Fazari et al., 2012). It is unclear whether *Minc-P1, Mare-P1*, and *Mjav-P1* on P1 are all part of a single gene with a broad spectrum of action, or whether they belong to separate genes forming a new cluster, as observed on P9 for *Me3, Me1*, and *N*, which have different spectra of action against RKN species (Hendy et al., 1985; Thies and Fery, 2000). However, our results provide two lines of evidence in support of these QTLs belonging to different genes within a cluster. Firstly, for the *Mare-P1* and *Mjav-P1* QTLs, the resistant YW allele displayed partial dominance, whereas partial dominance of the susceptible allele from DLL was observed for *Minc-P1*, suggesting different modes of action. Secondly, F_2_ individuals displaying genetic recombination between the markers at the peak values of the *Minc-P1* and *Mare-P1* loci were detected and the phenotypes of the corresponding F3 families confirmed recombination in the F2 plant, with a homozygous resistant or susceptible genotype at one QTL and a heterozygous genotype at the other QTL (**Table 3**). Despite their very tight linkage, these QTLs thus probably belong to different genes conferring different specificities against RKN populations. These findings provide further evidence that broad-spectrum quantitative resistance can result from the combination of race-specific resistance factors.

An additional major QTL, *Mjav-P9*, with a major dominant effect for resistance against *M. javanica*, was mapped to pepper chromosome P9, close to the B94 marker. The detection of a new resistance factor at this site was not unexpected, because the P9 genomic region also carries the *Me* and *N* genes (Fazari et al., 2012), together with the *R*-gene *Bs2*, which confers resistance to the bacterial pathogen *Xanthomonas campestris* pv. *Vesicatoria* (Mazourek et al., 2009), and QTLs for resistance to potyvirus PVY (Wang et al., 2008) and to the oomycete *Phytophthora capsici* (Thabuis et al., 2004). The genomic sequence of this P9 chromosomal region also has a high density of NBS–LRR genes from the *Bs2* subclass (82 genes), highlighting the “explosive expansion of the pepper genome” relative to those of other Solanaceae species (Kim et al., 2014). This expansion, which probably involved tandem duplications, resulted in diversification and a clustering of the *R*-genes on chromosome P9, and the *Mjav-P9* QTL, which has a mostly dominant effect, probably belongs to this cluster.

Broad-spectrum *R*-genes are often preferentially used in breeding programs, but previous studies have shown that the use of *R*-genes in an inappropriate genetic background may decrease their efficacy, in turn affecting their durability. The strategy of combining an *R*-gene with a partially resistant genetic background (i.e., a background carrying relevant QTLs) to increase its durability has been evaluated and validated in other pathosystems (Palloix et al., 2009; Brun et al., 2010; Fournet et al., 2013). Quenouille et al. (2012) suggested that this effect was due mostly to the additional resistance conferred by QTLs from the genetic background, decreasing the size of the pathogen population and, thus, the risk of emergence and of the further selection of virulent variants. For interactions between pepper and RKNs, YW proved to be a better genetic background than DLL for strengthening the efficacy of *Me1* or *Me3* (Barbary et al., 2014) and reducing the frequency of *Me3* resistance breakdown (Djian-Caporalino et al., 2014). However, the genetic determinants of this partial resistance had never been characterized. The new QTLs identified in this study (i.e., *Minc-P1, Mare-P1, Mjav-P1*, and *Mjav-P9*) are, thus, good candidates for pyramiding with *Me1, Me3*, or *N*, providing new opportunities for combining major and partial resistance factors together in pepper cultivars.

In terms of plant breeding, the location of the newly identified resistance factors on pepper chromosome P1 should facilitate their introgression by MAS, alongside current *R*-genes. Indeed, all the resistance genes effective against RKNs mapped to date are closely linked on pepper chromosome P9 (Fazari et al., 2012). However, they are in repulsion phases, with the different genes carried by different pepper accessions. *Minc-P1, Mare-P1, and Mjav-P1* are independent of this cluster and all are carried by the same accession (YW). This should make it easier to generate homozygous plant genotypes harboring resistance factors from both the P9 and P1 clusters. Breeders could also make use of *Minc-P1, Mare-P1*, and *Mjav-P1*, which do not act as fully dominant *R*-genes and may act through different resistance mechanisms. He et al. (2014) reported two QTLs affecting two different processes in RKN attack on cotton plants: root galling and egg production. These QTLs provided strong resistance to RKNs when combined in the same genotype. We are currently investigating this aspect in our pathosystem, by performing histological time-course studies to explore the spatiotemporal induction of plant responses conferred by these new resistance factors in pepper. In particular, the kinetics of J2 invasion in the roots, and the timing and location of cell necrosis (if any) will be investigated. On the basis of our preliminary observations, we hypothesize that the newly identified QTLs will induce defense reactions different from the classical HR triggered by the major *R*-genes *Me1* and *Me3*. We therefore anticipate that the successful combination of these qualitative and quantitative resistance factors into elite cultivars will provide new opportunities for enhanced and durable RKN resistance in pepper.

## Author Contributions

AB, PC-S, CD-C, AP, and BC conceived the project, contribute to technical work, to data treatment and article writing, AF and NM organized and performed technical work.

## Conflict of Interest Statement

The authors declare that the research was conducted in the absence of any commercial or financial relationships that could be construed as a potential conflict of interest.

## Footnotes

1 http://www.r-project.org/
