# Supplementary material for: Plant Genetic Background Increasing the Efficiency and Durability of Major Resistance Genes to Root-knot Nematodes Can Be Resolved into a Few Resistance QTLs
Source: Front Plant Sci. 2016 May 10;7:632. doi: 10.3389/fpls.2016.00632 (PMC4861812; doi:10.3389/fpls.2016.00632)
Supplement: Supplementary file 1 [file Data_Sheet_1.DOC]

**Supplementary data 1**: Genetic linkage map of pepper derived from *C. annuum* cv Yolo Wonder x *C. annuum* cv Doux Long des Landes F2 population of 130 F2 plants, based on 13 SSRs, 312 SNPs and 1 SCAR markers. On the left of each linkage group are indicated distances in centimorgans, calculated by the Kosambi mapping function. * indicates loci with segregation distortion. The positions of QTLs are presented on the right side of the linkage groups, in black for *M. incognita*, in blue for *M. arenaria*, and in green for *M. javanica*. *Me* genes cluster is figure in brown on chromosome 9. Bars represent confidence intervals associated with the resistance factor.

**Supplementary data 2**: Flanking sequences of the SNP markers flanking the confidence interval (LODmax-1) of the QTLs.

| Marker | QTL | Sequence |
| --- | --- | --- |
| SP1164 | Mare-P1 | TCGATGTGGAGTTTGTTGGCAAGGCTTAGATTTAAGCAGCAAAAAGTATA[G/C]TTTCTTCCATTGAGCTGTTCATGAAATGATTATTCTTCTTTGCAGCCAAT |
| SP1790 | Mare-P1 | GAGTCACGAGGTAAGGGATAAAATGGGTAAAACAGGGATGTCAGAAGAGT[A/G]TTATTGGTAGACTATTGTTCTTGTGTTCTCCCTTCAAGTGAATGAAACAA |
| SP1798 | Minc-P1 | CCTTATATAGNACANNCCAGCACGGATTAATTTCCCTAAGAAGAAAGGTG[G/A]ACTTTGGAGAATCAACAACATATATCTTTTGGCTTCGTTACTGCAGGTAG |
| SP1781 | Minc-P1 | GGTGATTTTGAGGCTGTCTATCTCCCACGTTTTATTCTTTGTGATCCCTA[A/G]TCTAGCTTGTGGTTGCAACNTCCACTTTCATTGTGTAACACCCTTGATCT |
| SP573 | Mjav-P1 | AAGACTTCATCTTTAGTCCGACGAAGGAGAAAAGGCATCACCTGTCAAGG[A/C]AAACCTTCCAGTTTGAATCTAGACGAGAAGAGATCCGAACTGTGAAGGCC |
| SP1781 | Mjav-P1 | GGTGATTTTGAGGCTGTCTATCTCCCACGTTTTATTCTTTGTGATCCCTA[A/G]TCTAGCTTGTGGTTGCAACNTCCACTTTCATTGTGTAACACCCTTGATCT |
| SP668 | Mjav-P9 | CAGCTGAGAGACGAGGGTCATATATCTCGATACAGCATCAAAGCAACACC[A/C]GGAGTCCAGGATTGTCTGCATTGGTGCTTACCCGGTGTTCCNNNNNNNNN |
| SP381 | Mjav-P9 | AATCATTCACACAGCATATTTTTTGTCGACCAATTTGCCAGTGCCTAGAC[A/G]TTCTGAATGAGGAGATCAAATCATTATCAGGACATGTATTTAGTCCCTTA |
